# A Rare Case of Large Skull Base Meningioma Mimicking Otitis Media with Effusion

**DOI:** 10.1155/2013/396805

**Published:** 2013-12-19

**Authors:** Musaed Alzahrani, Louis Gaboury, Issam Saliba

**Affiliations:** ^1^Division of Otorhinolaryngology, Head & Neck Surgery, Montreal University Hospital Center (CHUM), 1560 Rue Sherbrooke Est, Montréal, QC, Canada H2L 4M1; ^2^Department of Pathology and Cell Biology, University of Montreal, 6128 Succursale Centre-ville, Montréal, QC, Canada H3C 3J7

## Abstract

A 48-year-old woman presented with unilateral hearing loss and tinnitus for three years associated with middle ear effusion. Previous treatments, including antibiotics, corticoids, and transtympanic tube, were ineffective. Otomicroscopy showed a greyish retrotympanic mass associated with middle ear effusion. High resolution CT scan of the mastoid was in favor of chronic oto-mastoiditis without any evidence of tegmen dehiscence. Surgical exploration revealed a polypoid greyish mass filling the tympanic cavity. Histological examination postoperatively revealed a meningothelial meningioma. Postoperative magnetic resonance imaging (MRI) was obtained and showed a large skull base meningioma, extending from the clivus anteriorly to the porus acusticus posteriorly with middle ear invasion. After discussion with the multidisciplinary tumor board, it was managed by stereotactic radiotherapy due to the high surgical associated neurovascular risks. In conclusion, middle ear meningioma, although still a rare presentation, should be suspected in the presence of atypical chronic OME.

## 1. Introduction

Otitis media with effusion (OME) secondary to skull base meningioma is a rare condition and only few cases are reported in the literature [1]. In unilateral OME, flexible nasopharyngoscopy is a routine examination to rule out nasopharyngeal pathology. However, other causes like skull base meningioma can mimic OME. They present with classical OME symptoms as hearing loss, tinnitus, or vertigo [2].

In this paper, we present a case of a large skull base meningioma mimicking chronic OME. We also discuss its histological pattern, imaging characteristics, and the therapeutic modalities.

## 2. Case Report

A 48-year-old woman was referred to our tertiary care center for persistent right OME. Her main complaint was unilateral hearing loss associated with tinnitus. For the past three years, she has received multiple antibiotics courses without benefit. Transtympanic ventilation tube was inserted and spontaneously expulsed. She denied history of acute otitis media, chronic otorrhea, or vertigo.

Otomicroscopic examination of the left ear was normal. On the right side, the tympanic membrane (TM) was intact, slightly erythematous, and bulging. We decided to put a subannular tympanic tube under local anesthesia. However, after elevating the tympanomeatal flap, we noticed a greyish mass filling the mesotympanic cavity resembling cholesteatoma. The tube was inserted and the patient was scheduled for canal wall up mastoidectomy. Flexible nasopharyngoscopy examination was normal and there were no neurological deficits.

**Figure 1:**
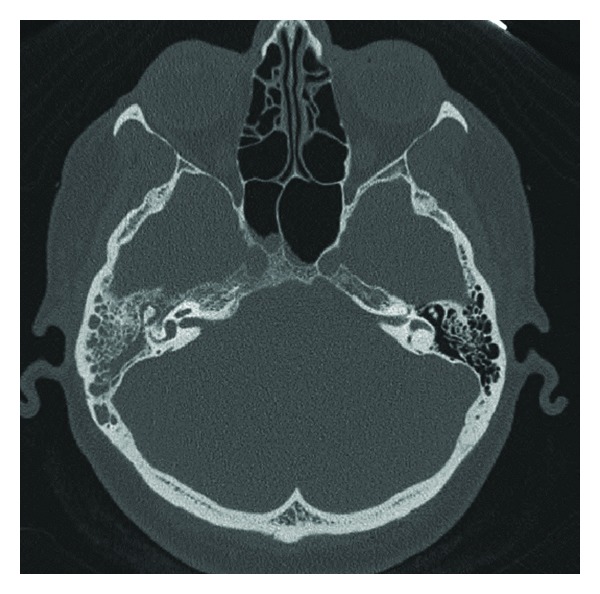
Axial high-resolution CT scan showing a right tympanomastoid homogenous opacity and petrous bone remodeling.

Audiometric studies showed a conductive hearing loss with an airbone gap of 30 dB HL. Axial high-resolution temporal bone CT scan demonstrated homogenous opacity of the mastoid and tympanic cavities without bone erosions (Figure 1).

Intraoperatively, a multilobulated greyish mass was filling the tympanic cavity and extending posteriorly to the antrum. The ossicular chain was intact and completely engulfed by the lesion rendering complete excision impossible. TM healing was excellent at 1 month postoperatively and there was no CSF otorrhea.

**Figure 2:**
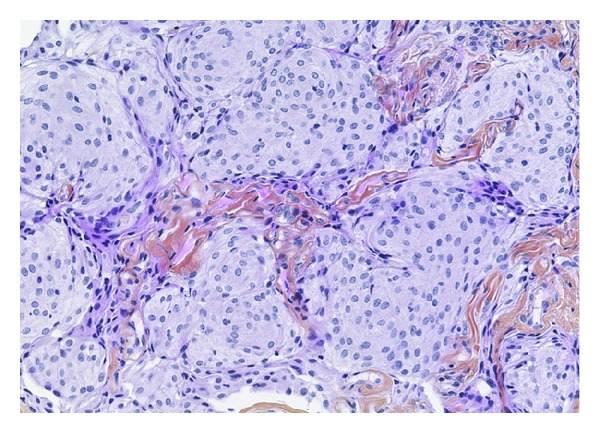
Haematoxylin and eosin staining showing an infiltrative growth pattern with whorled appearance.

Histologic studies confirmed the diagnosis of meningothelial meningioma. The lesion had an infiltrative growth pattern with whorled and nested neoplastic cells that display indistinct cell borders and round to oval nuclei with fine chromatin. Characteristic psammoma bodies and nuclear inclusions were also found (Figure 2).

**Figure 3:**
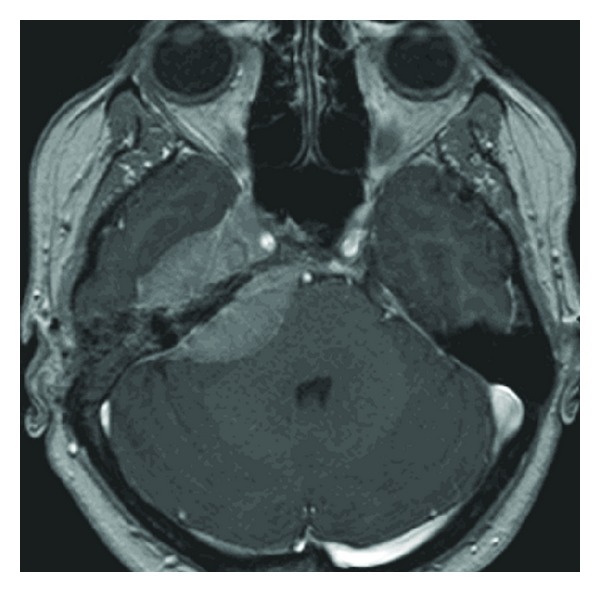
Axial, T1-weighted magnetic resonance imaging scans with gadolinium contrast, showing a right enhancing meningeal lesion involving the anterior and the posterior part the petrous apex with the characteristic meningeal tail.

MRI was then performed postoperatively and demonstrated a large skull base meningioma measuring 7 cm and extending from the clivus anteriorly to the porus acusticus posteriorly (Figure 3). Extension to the ME through the tegmen tympani was also identified.

A neurosurgical consultation was then solicited in addition to the radiooncology team. After discussion in the multidisciplinary tumor board, complete excision was judged to be difficult with high risks of neurovascular injuries and the resultant postoperative functional disabilities. So stereotactic radiation therapy was decided for this patient.

## 3. Discussion

Meningiomas are benign tumors, slow-growing originating from the arachnoid villi of the meninges [1]. Two forms of intracranial meningioma are described: (1) globular, the most common, and (2) en plaque. The majority of intracranial meningiomas are benign (90%) and less commonly atypical (6%) or malignant forms (2%) [3].

Extracranial meningioma accounts for less than two percent of meningiomas [4]. It was classified by Nager in 1966 into (1) type one representing extension of intracranial meningioma and (2) type two (Extracranial) occurring without intracranial extension [5].

ME extension of meningioma follows multiple pathways: the tegmen tympani, posterior fossa, the jugular foramen, and the internal auditory canal [2]. The presenting symptoms are similar to OME such as hearing loss, tinnitus, and dizziness and they are usually misdiagnosed for chronic OME.

MRI is the best imaging modality for the diagnosis of meningioma and typically shows an enhancing, dural-based, soft tissue mass with a characteristic enhanced dural tails. CT scan might show hyperostotic reactions [2] as well as bony defects on coronal reconstructions. However, the definitive diagnosis is obtained by histologic studies.

Microscopically they consist of small nests separated by strands of dense connective tissue. Each nest consists of polygonal cells with faintly acidophilic cytoplasm. Cell borders are typically indistinct resulting in a syncytial growth pattern. Nuclei are typically round or oval and contain inconspicuous nucleoli. There is no polymorphism, mitotic activity, nor tumor necrosis. Occasionally one may observe psammoma bodies. Immunohistochemical profiles include staining with EMA and vimentin. Histologically the differential diagnosis includes *Glomus* tumors and ceruminomas. However, the histological pattern is quite characteristic.

The treatment of choice for meningioma is surgical excision with an overall survival rate of 85%, 75%, and 70% at 5, 10, and 15 years, respectively [6]. However, total resection is not always feasible especially in large meningiomas, high neurovascular risks, or medically unfit patients and stereotactic radiotherapy is preferred. Although still controversial, radiotherapy is the standard treatment for atypical, recurrent, or malignant meningioma [6]. Meningioma was considered for long time as mostly radioresistant. Recent advances in radiation techniques have allowed the delivery of higher doses up to 60 Gy [6]. In such cases, neighboring organs, which might have a lower radiotherapy tolerance doses, are at risk of toxicity. However, there is a steep dose fall-off near the radiated lesion boundaries decreasing up to 30% per mm [6].

In conclusion, ME meningioma, although still a rare presentation, should be suspected in the presence of atypical chronic OME. MRI or biopsy through TM paracentesis, if possible, is advised to avoid unnecessary delay of diagnosis.
